# FOXA1 mutations in hormone-dependent cancers

**DOI:** 10.3389/fonc.2013.00020

**Published:** 2013-02-14

**Authors:** Jessica L. L. Robinson, Kelly A. Holmes, Jason S. Carroll

**Affiliations:** ^1^Cancer Research UK, Robinson WayCambridge, UK; ^2^Department of Oncology, University of CambridgeCambridge, UK

**Keywords:** FoxA1, breast cancer, prostate cancer, exome sequencing, SNP, androgen receptor (AR), estrogen receptor alpha

## Abstract

The forkhead protein, FOXA1, is a critical interacting partner of the nuclear hormone receptors, oestrogen receptor-α (ER) and androgen receptor (AR), which are major drivers of the two most common cancers, namely breast and prostate cancer. Over the past few years, progress has been made in our understanding of how FOXA1 influences nuclear receptor function, with both common and distinct roles in the regulation of ER or AR. Recently, another level of regulation has been described, with the discovery that FOXA1 is mutated in 1.8% of breast and 3–5% prostate cancers. In addition, a subset of both cancer types exhibit amplification of the genomic region encompassing the FOXA1 gene. Furthermore, there is evidence of somatic changes that influence the DNA sequence under FOXA1 binding regions, which may indirectly influence FOXA1-mediated regulation of ER and AR activity. These recent observations provide insight into the heterogeneity observed in ER and AR driven cancers.

Hormone-dependent breast and prostate cancers, constitute a major global cancer burden, in females and males, respectively, with a combined total of nearly 2.3 million new cases each year (Ferlay et al., 2010). In a large proportion of these cancers, the nuclear receptors AR in prostate cancer and ER in breast cancer, drive tumor growth in response to activation by their natural ligands, testosterone and oestrogen. A protein commonly expressed in hormonally-driven cancers is the transcription factor FOXA1 which appears to be intrinsic to tumor development in both breast and prostate cancer. Recent discoveries of amplification of the FOXA1 locus, mutations within the FOXA1 gene and mutations in the genomic regions FOXA1 occupies, are shedding light on mechanisms that perturb FOXA1 function and ultimately ER/AR activity.

## FOXA1 is critical for AR and ER function in cancer

**Figure 1:**
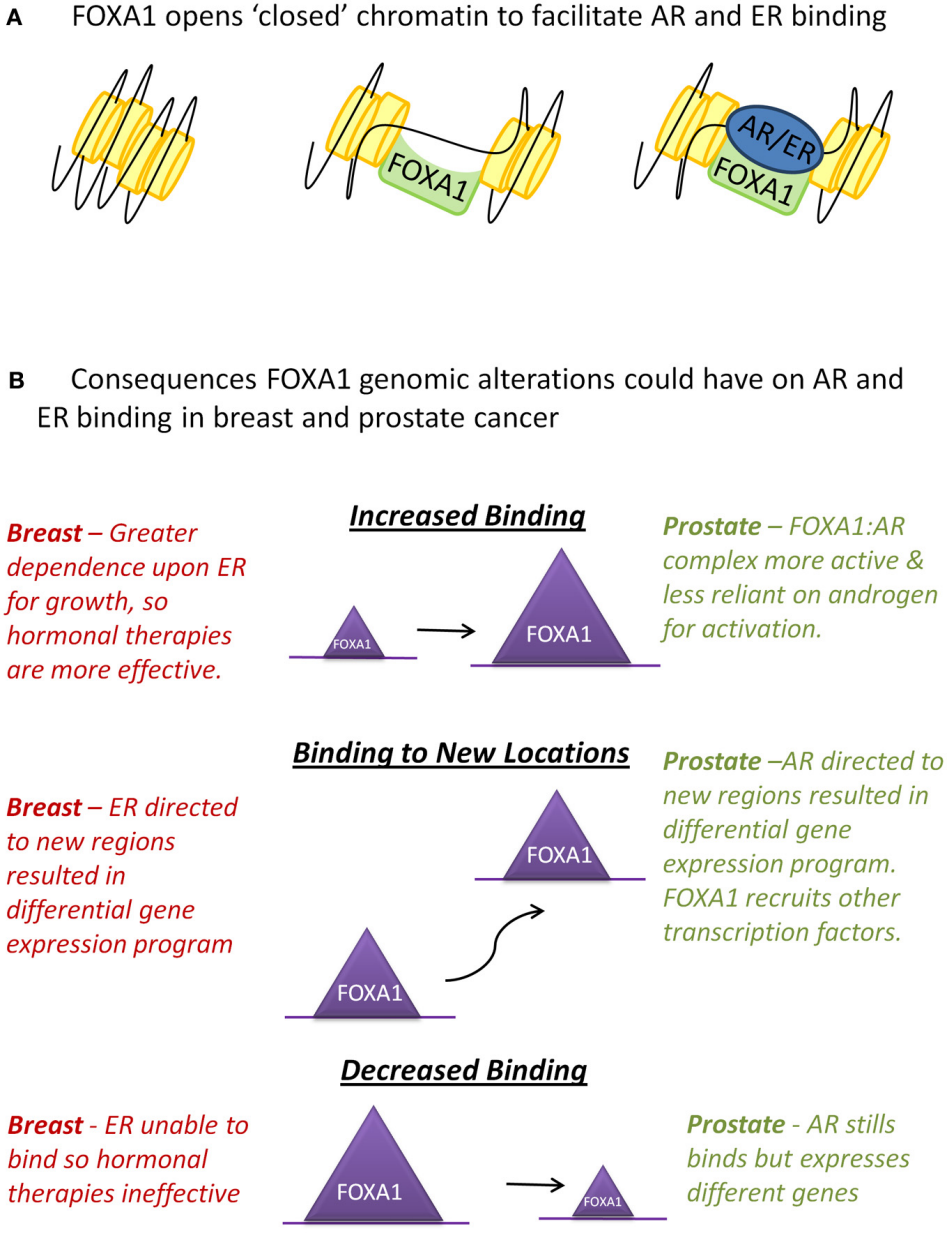
**Interactions between FOXA1 and the hormone receptors AR and ER. (A)** WT FOXA1 is able to bind to condensed chromatin and make this region more accessible to AR and ER which bind to DNA after hormone stimulation. **(B)** Various genomic alterations can occur to FOXA1 such as amplification of the FOXA1 locus, mutations to the coding sequence of FOXA1 or single nucleotide variants (SNVs) in the forkhead motif found at most FOXA1 binding events. This could results in an increase, decrease, or change in position of FOXA1 binding which in turn could affect AR and ER in the ways described above.

FOXA1 is one of three members of the highly related FOXA family. All forkhead proteins contain a “winged helix” DNA binding or forkhead domain, consisting of three α-helices, three β-sheets, and two loops or wings (Hannenhalli and Kaestner, 2009). The crystal structure of the forkhead domain shows that FOXA1 sits in the major groove of DNA with the loops making site-specific DNA contacts in a manner that closely resembles linker histone (Clark et al., 1993). FOXA proteins are often termed “pioneer factors” because they are able to bind to highly compacted, or “closed,” chromatin (Cirillo et al., 1998) and through their C-terminal domain make these genomic regions more accessible to other transcription factors (Cirillo et al., 2002) (Figure 1A).

Like other forkhead proteins, FOXA1 plays a key role in development, chiefly the lung and liver (Wan et al., 2004; Lee et al., 2005). With regards to the development of mammary glands and prostate rudiment, the *FoxA1^−/−^* mouse shows a normal phenotype at birth, but neither tissue is able to respond to hormone induction through AR and ER during puberty which results in ductal branching and epithelial cell maturation (Gao et al., 2005; Bernardo et al., 2010). FOXA1's direct interaction with AR and ER was first shown at the single locus level, probasin and PSA for AR (Gao et al., 2003), and vitellogenin for ER (Robyr et al., 2000). Later genome wide chromatin-immunoprecipitation experiments (ChIP-seq) for AR, ER, and FOXA1 in breast and prostate cancer cell lines and primary tumor tissue revealed a high level of co-occupancy between this pioneer factor and its respective nuclear receptor, presumably mediated by the forkhead motif found at AR and ER binding events (Carroll et al., 2006; Wang et al., 2007; Jia et al., 2008; Hurtado et al., 2011). Loss of FOXA1 expression by targeted siRNA transfection in breast and prostate cancer cell lines results in reduction of growth suggesting an essential role in the proliferation of both cancers (Hurtado et al., 2011; Robinson et al., 2011; Zhang et al., 2011) yet the mechanistic role for FOXA1 in ER and AR biology appears to be different.

FOXA1 is required for global ER binding in the MCF7 breast cancer cell line and ER occupancy at >90% of binding events is reduced when FOXA1 is silenced, which correlates with a global loss in the accessibility of chromatin (Hurtado et al., 2011). This loss of ER binding blocks ER-mediated gene expression and proliferation. In prostate cancer, however, when FOXA1 is lost, AR can still bind to chromatin and in fact occupies new binding sites which are coupled to changes in its' transcriptional targets (Sahu et al., 2011; Wang et al., 2011). Moreover, FOXA1 expression levels have opposing effects on patient outcome in breast and prostate cancer. In breast cancer, FOXA1 has been shown in multiple studies to be an independent marker for good outcome (Badve et al., 2007; Hisamatsu et al., 2012); most probably because the presence of FOXA1 indicates a functional ER complex which will respond well to anti-oestrogen compounds such as Tamoxifen. Conversely, in prostate cancer, high levels of FOXA1 correlate with poor prognosis (Sahu et al., 2011; Gerhardt et al., 2012). FOXA1 and AR levels correlate with one another, suggesting that an overactive AR transcriptional complex may be present in these tumors to overcome androgen deprivation more effectively. Interestingly, there is evidence of a common reliance in late stage disease as FOXA1 levels are high in both breast and prostate cancer metastases (Jain et al., 2011; Ross-Innes et al., 2012).

## Advances in DNA sequencing technologies

Genomic sequencing capacity has increased at an extraordinary rate, enhancing our ability to interrogate genetic changes and global genomic patterns. Since the first cancer genome was sequenced in 2008 (Ley et al., 2008), there have been numerous other sequencing studies with increasing numbers of tumors at greater genome coverage, made possible by improvements in DNA sequencing output coupled with a large decrease in cost (Meyerson et al., 2010). Whole exome- and whole-genome sequencing allow unbiased sequencing of cancer vs. normal exons or entire genomes to look for cancer specific somatic mutations and chromosomal rearrangements, amplifications or deletions. All the studies in this review have a minimum of 30-fold coverage over each gene, meaning it is possible to detect mutations which may only occur in a subset of cells in the tumor. Transcriptomic data from RNA-seq is also being mined to assess for changes in transcript expression levels that are influenced by these mutations. Integration of these genetic alterations will provide insight into the events that contribute to tumor progression.

## Mutations in prostate cancer

Unlike breast cancer, prostate cancer patients cannot be prognostically stratified based on mRNA expression profiles, therefore it is critical that genomic contributors to outcome are defined. Two large prostate cancer sequencing papers recently reported that FOXA1 mutations occur in 3.4–5.2% of tumors (Barbieri et al., 2012; Grasso et al., 2012). This finding has not been observed in any of the previous prostate cancer sequencing studies, probably because those earlier studies either lacked the number of samples or the sequencing depth required to detect variants that were present at low frequency (Taylor et al., 2010; Berger et al., 2011; Kumar et al., 2011).

The first study to report mutations in FOXA1 performed whole exome sequencing on 112 treatment-naive prostate adenocarcinomas as well as RNA-seq analysis on transcripts from 63 tumors (Barbieri et al., 2012). They found 12 genes which were significantly mutated, all of which were highly expressed in prostate cancer. A total of eight non-silent mutations in FOXA1 were identified clustered around the forkhead DNA binding domain (Table 1). Grasso et al., independently discovered by exome sequencing, FOXA1 to be mutated in a single tumor from their cohort of 11 high-grade localized prostate cancers but in none of their 50 metastatic castrate resistant prostate cancers (CRPC) obtained from rapid autopsies (Grasso et al., 2012). The mutation was a 2 bp insertion which resulted in a frame shift (S453fs) and two more frame shift mutations were detected in FOXA1 from exome sequencing of 11 prostate cancer cell lines (P358fs in hormone-sensitive LAPC-4 and A339fs in castrate resistance model DU-145). Based on this initial observation, a total of 101 localized prostate cancers and 46 CRPCs were subsequently targeted sequenced, within which five harbored mutations in FOXA1. Only one CRPC sample harbored a FOXA1 mutation which was isolated from a patient included in the initial exome study, but the DNA used in the targeted sequencing was obtained from a different site suggesting the FOXA1 mutation was a divergent event not seen in the primary tumor.

**Table 1:** **FOXA1 mutations in breast and prostate cancer**.

**Cancer**	**Sequencing method**	**Amino acid change**	**Type of mutation**	**Position in FoxA1**	**Occurrence (fraction of patients)**	**References**
Breast	Whole exome	A153V	Missense	N-terminal	1/507	TCGA, 2012
		S194fs	Frame shift	Forkhead domain	1/507	
		H247Y	Missense	Forkhead domain	1/507	
		***D226N***	Missense	Forkhead domain	1/507	
		S250F	Missense	Forkhead domain	1/507	
		I176M	Missense	Forkhead domain	2/507	
Prostate	Whole exome	F400I	Missense	C-Terminal TA domain	1/46 - CRPC	Grasso et al., 2012
		D226N^*^	Missense	Forkhead domain	1/111	Barbieri et al., 2012
		A232V	Missense	Forkhead domain	1/111	
		M253R^*^	Missense	Forkhead domain	1/111	
		M253K^*^	Missense	Forkhead domain	1/111	
	RNA-seq	M253R^*^	Missense	Forkhead domain	1/41	Barbieri et al., 2012
		M253K^*^	Missense	Forkhead domain	1/41	
		D226N^*^	Missense	Forkhead domain	1/41	
		D226Y	Missense	Forkhead domain	1/41	
	Sanger	G87R	Missense	N-Terminal TA domain	1/101	Grasso et al., 2012
		L388M	Missense	C-Terminal TA domain	1/101	
		S453fs	Frame shift	C-Terminal TA domain	1/101	
		L455M	Missense	C-Terminal TA domain	1/101	
		P358fs	Frame shift	C-Terminal TA domain	N/A - LAPC-4 Cell Line	
		A339fs	Frame shift	C-Terminal TA domain	N/A - DU-145 Cell Line	

TA, transactivation; CRPC, castrate resistant prostate cancer. Unless otherwise stated sample was extracted from a primary tumor. Barbieri et al. used two independent cohorts of patients for exome sequencing and RNA-seq therefore mutations seen by both techniques were identified in two independent patients (indicated by

* *). **D226N** is the only mutation seen in both breast and prostate cancer*.

## Mutations in breast cancer

Six major breast cancer genomic studies were published in 2012 which utilize high-throughput sequencing techniques to tease apart the important mutations, chromosomal rearrangements, and DNA methylation changes which occur in breast cancer. The findings have reinforced the role of known breast cancer driver genes such as TP53, ERBB2, (HER2) and PIK3CA and led to the discovery of a plethora of novel oncogenic mutations, including FOXA1 in The Cancer Genome Atlas (TCGA) study (TCGA, 2012). The TCGA study assessed a total of 825 patients to ranging extents on six different platforms, including exome sequencing of 507 tumors and matched normal DNA. They identified eight tumors with mutations in FOXA1 (Table 1) placing it on the cusp of being a significantly mutated gene as determined by the MuSiC package (Dees et al., 2012). In total, they found 35 genes significantly mutated and two genes at borderline significance (FOXA1 and CTCF). This raises an important point because none of the other four studies which carried out whole-exome or whole-genome sequencing reported any FOXA1 mutations (Banerji et al., 2012; Ellis et al., 2012; Shah et al., 2012; Stephens et al., 2012). These studies all had smaller sample sizes (ranging from 77 to 108 samples), which would not have been sufficient to detect FOXA1 mutants which, based on the TCGA study, only occur in 1.8% patients. Furthermore, one of the studies (Shah et al., 2012) used a selected population of exclusively ER-PR-HER2-patients and all FOXA1 mutations in the TCGA study were found in ER+ tumors.

Gene expression profiling was applied to the eight tumors identified with FOXA1 mutations and the majority were luminal A tumors (5/8) and the remainder were luminal B (2/8) and HER2 (1/8). Another ER interacting partner, GATA3, was found to be highly mutated in all the studies (10.8% of breast cancers in TCGA study), although GATA3 and FOXA1 mutations were mutually exclusive.

## FOXA1 copy number alterations

The genomic region encompassing the FOXA1 gene (14q21.1) is amplified in a range of cancers (Yasui et al., 2001; Lin et al., 2002; Nucera et al., 2009; Deutsch et al., 2012) and there is increasing evidence this also occurs in prostate and breast cancer. FOXA1 amplification in prostate cancer was identified from the profiling of eight systemic metastatic tumors in a range of organs from six unrelated patients. Focal amplification of 14q21.1 was found in three independent lesions isolated from a single patient, suggesting amplification of the FOXA1 genomic region is an early event in progression of this tumor (Robbins et al., 2011). Furthermore, there is evidence of amplification of FOXA1 in a lymph node metastasis in an independent cohort (Grasso et al., 2012).

In breast cancer, the TCGA study reported that 1% of the 773 breast cancer tumors tested have focal amplification of the region containing the FOXA1 genomic locus, consisting of six ER+ and two HER2 tumors (TCGA, 2012). However, there was no evidence of amplification of this region in the larger study published by Curtis et al. which categorized 1992 breast cancers on the basis of copy number changes and mRNA profiles (Curtis et al., 2012) even though this study generally correlates well with the TCGA data.

## Sequence changes of the DNA binding motif modulate FOXA1 binding

FOXA1 binds primarily at enhancer regions of genes throughout the genome most of which contain the consensus forkhead (FKH) motif. Recently, a report described that single nucleotide polymorphisms (SNPs) in the binding sites of forkhead proteins can not only modulate FOXA1 binding to the chromatin, but lead to changes in expression of adjacent genes (Cowper-Sal Lari et al., 2012). Cowper-Sal Lari et al. developed a novel computational method called intragenomic replicates (IGR) to accurately predict the affinity of a transcription factor for the reference and variant alleles of a SNP in a given genomic context. Using ChIP-seq data for FOXA1 they are able to measure the affinity of FOXA1 for a given DNA sequence. They demonstrate that FOXA1 has a high affinity for DNA with a forkhead motif comprising of a C at position 8 combined with an A at position 6, a sequence combination which has six times greater affinity than when the base at position 6 is a G. This emphasizes the concept that transcription factor binding is not a binary event and that quantitative differences in binding can have a profound effect on activity. In the example provided, a base pair change at position 8 of the FKH motif occurs 18 kb upstream of the TOX3 gene with the variant SNP causing an increase in FOXA1 binding and a concomitant decrease in expression of TOX3, leading to increased cell proliferation.

Another report from the Lupien lab (Zhang et al., 2012) shows functional analysis of a SNP present in a prostate cancer risk locus which changes the tenth position of the FKH DNA motif. Using an *in vitro* reporter assay they show a 2-fold decrease in FOXA1 binding to the variant allele compared to the reference allele, resulting in a 50% increase in luciferase activity. Further supporting this work, a subset of SNPs occurring in hepatocellular carcinoma (HCC) can alter the binding of FOXA2, another forkhead protein, resulting in altered expression, a finding that was confirmed in patient samples on several target genes (Li et al., 2012). These examples point to the role that genetic variants can play on FOXA1-DNA interactions and the physiological impact that can have by changing expression levels of key target genes.

## Functional relevance of FOXA1 aberrations

Mutations in the coding sequence of FOXA1 occur in two clusters (Table 1), either in or around the forkhead DNA binding domain or in the C-terminal transactivation domain. Two mutational hotspots are present in the forkhead domain; position D226, mutated in a total of five patients, across both breast and prostate cancer, and position M253, mutated in four prostate tumors. Experimental modeling of these mutations within the DNA binding domain is yet to be conducted therefore it is not known whether they increase or perturb FOXA1 binding. However, previous studies on FOXA1 provide clues as to what may be the downstream effects of these changes.

In breast cancer if these mutations do diminish the binding capability of FOXA1, ER's dependency upon FOXA1 for binding (Hurtado et al., 2011) suggests that these tumors may have evolved to become independent of oestrogen for growth. These tumors would likely be more aggressive as they would not respond to standard hormonal therapies (Figure 1B). On the contrary, in prostate cancer, as AR is still able to bind in the absence of FOXA1, a mutation in FOXA1 which inhibits its DNA binding capacity may not inhibit AR binding, but could instead alter its binding profile and transcriptional targets (Sahu et al., 2011; Wang et al., 2011).

Alternatively, mutations within the forkhead domain could affect FOXA1's affinity for its canonical binding site, potentially resulting in binding to new genomic locations, which may result in substantial effects on the transcriptional program within that tumor, for example FOXA1's recruitment to novel sites in the CRPC cell line LNCaP-abl, allows it to become androgen independent (Zhang et al., 2011). FOXA1-DNA associations may also be affected on a site by site basis, due to SNPs occurring in FOXA1 binding sites (Cowper-Sal Lari et al., 2012). Insights into how changes in specific forkhead motifs can affect transcription factor recruitment at that site, as well as downstream gene expression changes, could prove to be essential in determining the “most important” FOXA1 binding events in cancer.

Grasso et al. conducted the first set of functional analysis of the N- and C-terminal mutations detected, by creating prostate cancer cell lines stably expressing five of the mutants (Grasso et al., 2012). In these preliminary experiments, only one of the mutations, L388M, caused a growth increase over wild type (WT) FOXA1 overexpression. AR-FOXA1 interaction was still important in this mutant cell line as it remained reliant upon androgen for growth. The S453fs mutant and WT FOXA1 were overexpressed in xenograft tumors which resulted in significantly larger tumors, but the mutation posed no growth advantage over WT. Even though the authors themselves express the need for further characterization of the mutations, we could speculate that this cluster of mutations within the C-terminus may change the constituents of the FOXA1 complex. In CRPC, AR splice variants are expressed which could preferentially bind to FOXA1 mutants, a feasible theory as these splice events often occur in the hinge domain of AR, which constitutes its' interaction point with FOXA1 (Wang et al., 2011).

Finally, FOXA1 binding may be increased due to a mutation stabilizing the protein via changes in its' DNA interaction affinity or through copy number gains in the FOXA1 locus resulting in elevated protein levels. In prostate cancer, FOXA1 genomic locus amplification was only observed in metastatic samples (Robbins et al., 2011; Grasso et al., 2012), which correlates with the observation of elevated FOXA1 staining in metastases and high grade tumors (Gerhardt et al., 2012). Increased levels of WT FOXA1 significantly increase prostate cancer proliferation and the size of xenograft tumors (Grasso et al., 2012), possibly due to increased AR-mediated growth. In breast cancer, however, high levels of FOXA1 are a marker of good prognosis (Badve et al., 2007; Hisamatsu et al., 2012) possibly because elevated FOXA1 in breast cancer engenders a greater reliance upon ligand-dependent ER activity to drive tumorigenesis, which in turn would generate hypersensitivity to anti-oestrogen therapies. This theory suggests that breast tumors with amplification of FOXA1 would be more responsive to treatment and the patients would have a better prognosis (Figure 1B).

Reliance upon ligands for nuclear receptor activation could be a fundamental difference between breast and prostate cancer and may explain why the two hormone receptors respond differently to gain and loss of FOXA1. If AR driven prostate cancers readily acquire ligand independence, elevated FOXA1-AR activity would be an advantage to the tumor, supporting the observation that high FOXA1 is a marker of poor patient outcome. In contrast, breast cancers may retain ligand dependence for longer, permitting the paradoxical acquisition of both elevated estrogen-mediated ER-FOXA1 activity and increased sensitivity to drugs that block this pathway. It will be critical to determine the key ER and AR target gene changes at high and low FOXA1 levels and any differences in the associated AR/ER protein complexes.

## Conclusion

It is evident that FOXA1 can be regulated at a genomic level either through somatic mutations, genomic amplification, or changes to the sequence within binding regions. Due to the lack of current follow up and low frequency of events there is no current indication on how these changes affect patient outcome. As such, it is important to collect functional data on the genomic changes observed in breast and prostate cancer.

### Conflict of interest statement

The authors declare that the research was conducted in the absence of any commercial or financial relationships that could be construed as a potential conflict of interest.

## References

[B1] Badve S. Turbin D. Thorat M. A. Morimiya A. Nielsen T. O. Perou C. M. (2007). FOXA1 expression in breast cancer – correlation with luminal subtype A and survival. Clin. Cancer Res. 13, 4415–4421 10.1158/1078-0432.CCR-07-0122 17671124

[B2] Banerji S. Cibulskis K. Rangel-Escareno C. Brown K. K. Carter S. L. Frederick A. M. (2012). Sequence analysis of mutations and translocations across breast cancer subtypes. Nature 486, 405–409 10.1038/nature11154 22722202 PMC4148686

[B3] Barbieri C. E. Baca S. C. Lawrence M. S. Demichelis F. Blattner M. Theurillat J.-P. (2012). Exome sequencing identifies recurrent SPOP, FOXA1 and MED12 mutations in prostate cancer. Nat. Genet. 44, 685–689 10.1038/ng.2279 22610119 PMC3673022

[B4] Berger M. F. Lawrence M. S. Demichelis F. Drier Y. Cibulskis K. Sivachenko A. Y. (2011). The genomic complexity of primary human prostate cancer. Nature 470, 214–220 10.1038/nature09744 21307934 PMC3075885

[B5] Bernardo G. M. Lozada K. L. Miedler J. D. Harburg G. Hewitt S. C. Mosley J. D. (2010). FOXA1 is an essential determinant of ERα expression and mammary ductal morphogenesis. Development 137, 2045–2054 10.1242/dev.043299 20501593 PMC2875844

[B6] Carroll J. S. Meyer C. A. Song J. Li W. Geistlinger T. R. Eeckhoute J. (2006). Genome-wide analysis of estrogen receptor binding sites. Nat. Genet. 38, 1289–1297 10.1038/ng1901 17013392

[B7] Cirillo L. A. Lin F. R. Cuesta I. Friedman D. Jarnik M. Zaret K. S. (2002). Opening of compacted chromatin by early developmental transcription factors HNF3 (FoxA) and GATA-4. Mol. Cell 9, 279–289 10.1016/S1097-2765(02)00459-8 11864602

[B8] Cirillo L. A. McPherson C. E. Bossard P. Stevens K. Cherian S. Shim E. Y. (1998). Binding of the winged-helix transcription factor HNF3 to a linker histone site on the nucleosome. EMBO J. 17, 244–254 10.1093/emboj/17.1.244 9427758 PMC1170375

[B9] Clark K. L. Halay E. D. Lai E. Burley S. K. (1993). Co-crystal structure of the HNF-3/fork head DNA-recognition motif resembles histone H5. Nature 364, 412–420 10.1038/364412a0 8332212

[B10] Cowper-Sal Lari R. Zhang X. Wright J. B. Bailey S. D. Cole M. D. Eeckhoute J. (2012). Breast cancer risk-associated SNPs modulate the affinity of chromatin for FOXA1 and alter gene expression. Nat. Genet. 44, 1191–1198 10.1038/ng.2416 23001124 PMC3483423

[B11] Curtis C. Shah S. P. Chin S.-F. Turashvili G. Rueda O. M. Dunning M. J. (2012). The genomic and transcriptomic architecture of 2,000 breast tumours reveals novel subgroups. Nature 486, 346–352 10.1038/nature10983 22522925 PMC3440846

[B12] Dees N. D. Zhang Q. Kandoth C. Wendl M. C. Schierding W. Koboldt D. C. (2012). MuSiC: identifying mutational significance in cancer genomes. Genome Res. 22, 1589–1598 10.1101/gr.134635.111 22759861 PMC3409272

[B13] Deutsch L. Wrage M. Koops S. Glatzel M. Uzunoglu F. G. Kutup A. (2012). Opposite roles of FOXA1 and NKX2-1 in lung cancer progression. Genes Chromosomes Cancer 51, 618–629 10.1002/gcc.21950 22383183

[B14] Ellis M. J. Ding L. Shen D. Luo J. Suman V. J. Wallis J. W. (2012). Whole-genome analysis informs breast cancer response to aromatase inhibition. Nature 486, 353–360 10.1038/nature11143 22722193 PMC3383766

[B15] Ferlay J. Shin H.-R. Bray F. Forman D. Mathers C. Parkin D. M. (2010). Estimates of worldwide burden of cancer in 2008: GLOBOCAN (2008). Int. J. Cancer 127, 2893–2917 10.1002/ijc.25516 21351269

[B16] Gao N. Ishii K. Mirosevich J. Kuwajima S. Oppenheimer S. R. Roberts R. L. (2005). Forkhead box A1 regulates prostate ductal morphogenesis and promotes epithelial cell maturation. Development 132, 3431–3443 10.1242/dev.01917 15987773

[B17] Gao N. Zhang J. Rao M. A. Case T. C. Mirosevich J. Wang Y. (2003). The role of hepatocyte nuclear factor-3a (Forkhead Box A1) and androgen receptor in transcriptional regulation of prostatic genes. Mol. Endocrinol. 17, 1484–1507 10.1210/me.2003-0020 12750453

[B18] Gerhardt J. Montani M. Wild P. Beer M. Huber F. Hermanns T. (2012). FOXA1 promotes tumor progression in prostate cancer and represents a novel hallmark of castrate-resistant prostate cancer. Am. J. Pathol. 180, 848–861 10.1016/j.ajpath.2011.10.021 22138582

[B19] Grasso C. S. Wu Y.-M. Robinson D. R. Cao X. Dhanasekaran S. M. Khan A. P. (2012). The mutational landscape of lethal castration-resistant prostate cancer. Nature 487, 239–243 10.1038/nature11125 22722839 PMC3396711

[B20] Hannenhalli S. Kaestner K. H. (2009). The evolution of Fox genes and their role in development and disease. Nat. Rev. Genet. 10, 233–240 10.1038/nrg2523 19274050 PMC2733165

[B21] Hisamatsu Y. Tokunaga E. Yamashita N. Akiyoshi S. Okada S. Nakashima Y. (2012). Impact of FOXA1 expression on the prognosis of patients with hormone receptor-positive breast cancer. Ann. Surg. Oncol. 19, 1145-1152 10.1245/s10434-011-2094-4 21984487

[B22] Hurtado A. Holmes K. A. Ross-Innes C. S. Schmidt D. Carroll J. S. (2011). FOXA1 is a key determinant of estrogen receptor function and endocrine response. Nat. Genet. 43, 27–33 10.1038/ng.730 21151129 PMC3024537

[B23] Jain R. K. Mehta R. J. Nakshatri H. Idrees M. T. Badve S. S. (2011). High-level expression of forkhead-box protein A1 in metastatic prostate cancer. Histopathology 58, 766–772 10.1111/j.1365-2559.2011.03796.x 21401706

[B24] Jia L. Berman B. P. Jariwala U. Yan X. Cogan J. P. Walters A. (2008). Genomic androgen receptor-occupied regions with different functions, defined by histone acetylation, coregulators and transcriptional capacity. PLoS ONE 3:e3645 10.1371/journal.pone.0003645 18997859 PMC2577007

[B25] Kumar A. White T. A. Mackenzie A. P. Clegg N. Lee C. Dumpit R. F. (2011). Exome sequencing identifies a spectrum of mutation frequencies in advanced and lethal prostate cancers. Proc. Natl. Acad. Sci. 108, 17087–17092 10.1073/pnas.1108745108 21949389 PMC3193229

[B26] Lee C. S. Friedman J. R. Fulmer J. T. Kaestner K. H. (2005). The initiation of liver development is dependent on Foxa transcription factors. Nature 435, 944–947 10.1038/nature03649 15959514

[B27] Ley T. J. Mardis E. R. Ding L. Fulton B. McLellan M. D. Chen K. (2008). DNA sequencing of a cytogenetically normal acute myeloid leukaemia genome. Nature 456, 66–72 10.1038/nature07485 18987736 PMC2603574

[B28] Li Z. Tuteja G. Schug J. Kaestner K. H. (2012). Foxa1 and Foxa2 are essential for sexual dimorphism in liver cancer. Cell 148, 72–83 10.1016/j.cell.2011.11.026 22265403 PMC3266536

[B29] Lin L. Miller C. T. Contreras J. I. Prescott M. S. Dagenais S. L. Wu R. (2002). The hepatocyte nuclear factor 3a gene, HNF3a (FOXA1), on chromosome band 14q13 is amplified and overexpressed in esophageal and lung adenocarcinomas. Cancer Res. 62, 5273–5279 12234996

[B30] Meyerson M. Gabriel S. Getz G. (2010). Advances in understanding cancer genomes through second-generation sequencing. Nat. Rev. Genet. 11, 685–696 10.1038/nrg2841 20847746

[B31] Nucera C. Eeckhoute J. Finn S. Carroll J. S. Ligon A. H. Priolo C. (2009). FOXA1 is a potential oncogene in anaplastic thyroid carcinoma. Clin. Cancer Res. 15, 3680–3689 10.1158/1078-0432.CCR-08-3155 19470727

[B32] Robbins C. M. Tembe W. A. Baker A. Sinari S. Moses T. Y. Beckstrom-Sternberg S. (2011). Copy number and targeted mutational analysis reveals novel somatic events in metastatic prostate tumors. Genome Res. 21, 47–55 10.1101/gr.107961.110 21147910 PMC3012925

[B33] Robinson J. L. L. Macarthur S. Ross-Innes C. S. Tilley W. D. Neal D. E. Mills I. G. (2011). Androgen receptor driven transcription in molecular apocrine breast cancer is mediated by FoxA1. EMBO J. 30, 3019–3027 10.1038/emboj.2011.216 21701558 PMC3160190

[B34] Robyr D. Gegonne A. Wolffe A. P. Wahli W. (2000). Determinants of vitellogenin B1 promoter architecture. J. Biol. Chem. 275, 28291–28300 10.1074/jbc.M002726200 10854430

[B35] Ross-Innes C. S. Stark R. Teschendorff A. E. Holmes K. A. Ali H. R. Dunning M. J. (2012). Differential oestrogen receptor binding is associated with clinical outcome in breast cancer. Nature 481, 389–393 10.1038/nature10730 22217937 PMC3272464

[B36] Sahu B. Laakso M. Ovaska K. Mirtti T. Lundin J. Rannikko A. (2011). Dual role of FoxA1 in androgen receptor binding to chromatin, androgen signalling and prostate cancer. EMBO J. 30, 3962–3976 10.1038/emboj.2011.328 21915096 PMC3209787

[B37] Shah S. P. Roth A. Goya R. Oloumi A. Ha G. Zhao Y. (2012). The clonal and mutational evolution spectrum of primary triple-negative breast cancers. Nature 486, 395–399 10.1038/nature10933 22495314 PMC3863681

[B38] Stephens P. J. Tarpey P. S. Davies H. Van Loo P. Greenman C. Wedge D. C. (2012). The landscape of cancer genes and mutational processes in breast cancer. Nature 486, 400–404 10.1038/nature11017 22722201 PMC3428862

[B39] Taylor B. S. Schultz N. Hieronymus H. Gopalan A. Xiao Y. Carver B. S. (2010). Integrative genomic profiling of human prostate cancer. Cancer Cell 18, 11–22 10.1016/j.ccr.2010.05.026 20579941 PMC3198787

[B40] TCGA. (2012). Comprehensive molecular portraits of human breast tumours. Nature 490, 61–70 10.1038/nature11412 23000897 PMC3465532

[B41] Wan H. Kaestner K. H. Ang S.-L. Ikegami M. Finkelman F. D. Stahlman M. T. (2004). Foxa2 regulates alveolarization and goblet cell hyperplasia. Development 131, 953–964 10.1242/dev.00966 14757645

[B42] Wang D. Garcia-Bassets I. Benner C. Li W. Su X. Zhou Y. (2011). Reprogramming transcription by distinct classes of enhancers functionally defined by eRNA. Nature 474, 390–394 10.1038/nature10006 21572438 PMC3117022

[B43] Wang Q. Li W. Liu X. S. Carroll J. S. Janne O. A. Keeton E. K. (2007). A hierarchical network of transcription factors governs androgen receptor-dependent prostate cancer growth. Mol. Cell 27, 380–392 10.1016/j.molcel.2007.05.041 17679089 PMC3947890

[B44] Yasui K. Imoto I. Fukuda Y. Pimkhaokham A. Yang Z.-Q. Naruto T. (2001). Identification of target genes within an amplicon at 14q12-q13 in esophageal squamous cell carcinoma. Genes Chromosomes Cancer 32, 112–118 10.1002/gcc.1172 11550278

[B45] Zhang C. Wang L. Wu D. Chen H. Chen Z. Thomas-Ahner J. M. (2011). Definition of a FoxA1 Cistrome that is crucial for G1 to S-phase cell-cycle transit in castration-resistant prostate cancer. Cancer Res. 71, 6738–6748 10.1158/0008-5472.CAN-11-1882 21900400 PMC4081454

[B46] Zhang X. Cowper-Sala Lari R. Bailey S. D. Moore J. H. Lupien M. (2012). Integrative functional genomics identifies an enhancer looping to the SOX9 gene disrupted by the 17q24.3 prostate cancer risk locus. Genome Res. 22, 1437–1446 10.1101/gr.135665.111 22665440 PMC3409257

